# Municipal health expectancy in Japan: decreased healthy longevity of older people in socioeconomically disadvantaged areas

**DOI:** 10.1186/1471-2458-5-65

**Published:** 2005-06-14

**Authors:** Yoshiharu Fukuda, Keiko Nakamura, Takehito Takano

**Affiliations:** 1 Health Promotion/International Health, Division of Public Health, Graduate School of Tokyo Medical and Dental University, 1-5-45 Yushima, Bunkyo-ku, Tokyo 113-8519, Japan

## Abstract

**Background:**

Little is known about small-area variation in healthy longevity of older people and its socioeconomic correlates. This study aimed to estimate health expectancy at 65 years (HE65) at the municipal level in Japan, and to examine its relation to area socio-demographic conditions.

**Methods:**

HE65 of municipalities (N = 3361) across Japan was estimated by a linear regression formula with life expectancy at 65 years and the prevalence of those certificated as needing nursing care. The relation between HE65 and area socio-demographic indicators was examined using correlation coefficients.

**Results:**

The estimated HE65 (years) ranged from 13.13 to 17.39 for men and from 14.84 to 20.53 for women. HE65 was significantly positively correlated with the proportion of elderly and per capita income, and negatively correlated with the percentage of households of a single elderly person, divorce rate, and unemployment rate. These relations were stronger in large municipalities (with a population of more than 100,000) than in small and medium-size municipalities.

**Conclusion:**

A decrease in healthy longevity of older people was associated with a higher percentage of households of a single elderly person and divorce rate, and lower socioeconomic conditions. This study suggests that older people in urban areas are susceptible to socio-demographic factors, and a social support network for older people living in socioeconomically disadvantaged conditions should be encouraged.

## Background

The ageing of the population has been progressing throughout the world, and Japan is the leading country with population ageing: the proportion of elderly (aged 65 years or over) was 17.4% in 2000 and is estimated to be over 35% in 2050 [1]. The quality of the years lived and well-being in an ageing society with declining mortality and increased life expectancy (LE) have been addressed [2].

To measure health levels including the aspects of quality of life and well-being, several comprehensive health indicators have been developed, and among them health expectancy (HE) has been commonly used [2-5]. HE is calculated by application of prevalence of morbidity data such as disability prevalence on age-specific person years in a life table [2,6]. The national health plan in Japan, "Health Japan 21", accompanied by local action plans, aims to prolong HE at the national and local levels through disease prevention and health promotion [7]. However, it is difficult to calculate and monitor HE for health policy planning and evaluation, especially at the local level, because of a lack of data on morbidity. Although previous studies have calculated HE at the prefectural level in Japan, these calculations used inconsistent and various databases of disability prevalence, and could not be applied at the municipal level because of limited availability of data [8-10].

In Japan, long-term care insurance (LTCI) was introduced in 2000 for older people needing nursing care, and citizens aged 40 years or over can receive insurance benefits after application and certification that they need nursing care. Since LTCI unions are managed by municipalities, it is theoretically possible to calculate HE using the municipal data of LTCI as morbidity. However, municipal HE has been calculated in only one prefecture [11], because the sex- and age-specific prevalence of those certificated as needing nursing care for LTCI by municipalities is not routinely published.

It was confirmed by previous studies that socio-demographic factors influence mortality, morbidity, and other health conditions of older people [12-15]. Socioeconomic status such as education level, occupational social class, and income has been demonstrated to be a critical factor predicting the health status of older people [13,15]. Family structure and living arrangement have been elicited as other critical factors influencing the health of older people, and conditions such as living alone and being widowed/divorced are related to poorer health status [16,17].

There was little relation between HE and socio-demographic indicators related to income and family structure at the prefectural level in Japan [18], while a municipal-level study in one prefecture showed a weak but significant relation between HE and socio-demographic indicators such as the percentage of nuclear households [11]. The relation between health status and regional characteristics might have been obscured due the higher level analysis which included heterogeneous smaller level regions, as a previous study demonstrated that the relation differed according to the area classification by urban-rural difference [19]. Analysis at different levels could lead to an inconsistent relation, and the smaller-area analysis more directly reflects the relation at the individual and household levels [20,21]. Therefore, studies examining the relation between socio-demographic factors and health including not only death but also morbidity at a smaller level will provide reliable evidence of the influence of socio-demographic factors on health in older people.

The purposes of this study were to estimate HE of older people at the municipal level across Japan, and to elucidate the association between HE and area socio-demographic factors.

## Methods

### Study units

**Figure 1:**
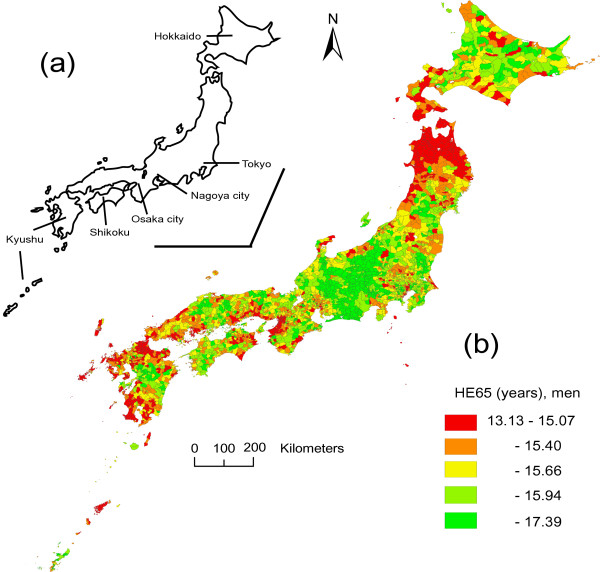
**Mapping of health expectancy for men**. Map of Japan (a) and health expectancy at 65 years (HE65) of men by municipality (b). Municipalities (N = 3361) are classified into quintiles according to HE65, and are colored accordingly.

**Figure 2:**
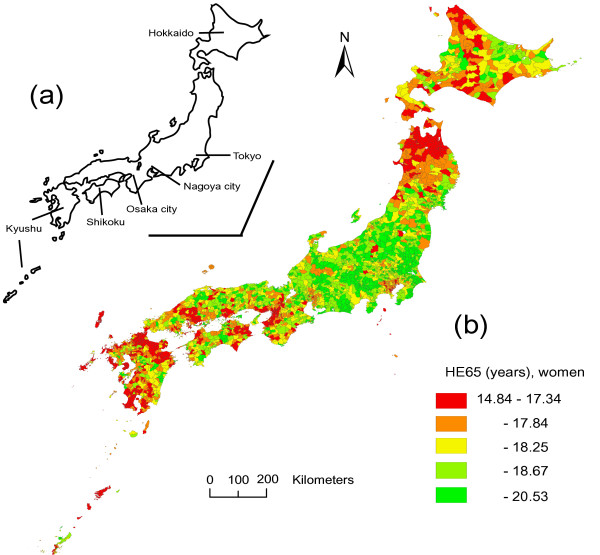
**Mapping of health expectancy for women**. Map of Japan (a) and health expectancy at 65 years (HE65) of women by municipality (b). Municipalities (N = 3361) are classified into quintiles according to HE65, and are colored accordingly.

Japan consists mainly of four islands (a map is shown in Figures 1 and 2). Hokkaido Island is located in the northern part, and includes only one prefecture (Hokkaido prefecture). The mainland (Honshu) includes several metropolitan areas such as Tokyo, Nagoya, and Osaka. The other islands are Shikoku and Kyushu.

According to Local Autonomy Law, local public entities in Japan are divided into two categories. The first category consists of cities, towns, and villages. The second category consists of prefectures (N = 47). All districts in the country belong to one of the municipalities and at the same time fall within the boundaries of one of the prefectures. As specific cases, the Tokyo prefecture (Tokyo Metropolis) includes 23 special wards ("ku") in addition to cities, towns and villages, and 12 large cities ("cities designated by ordinance") such as Nagoya and Osaka consist of wards ("ku"). In total, there were 3361 units of cities, towns and villages as well as Tokyo special wards and wards of cities designated by ordinance in December 2001.

### Estimation of health expectancy

Calculation of HE by the Sullivan method requires life-table data and the sex- and age-specific morbidity [6]. However, municipal data of the sex- and age-specific number of those certificated as needing nursing care for LTCI are not available, and only the total number was available by insurance union. Therefore, this study estimated HE at 65 years (HE65) by a linear regression formula using life expectancy at 65 years (LE65) and the certificated rate in LTCI (P): HE65 = a + b × LE65 + c × P.

The formula was drawn from prefectural data (N = 47) of precise HE65, which was calculated by the Sullivan method with the prefectural life-table in 2000 [22] and the sex- and age-specific certification rate of LTCI in 2002 [23]. The coefficient of determinant (R^2^) for the regression with LE65 and the crude certification rate was 0.913 for men and 0.906 for women. When the standardized certification ratio (SCR), which was age-standardised by the indirect method, was used as a predictor, R^2 ^was increased to 0.986 for men and 0.982 for women.

The validity of this estimation was confirmed by the municipal data (N = 59) of one prefecture (Shimane prefecture), in which the precise HE65 had been calculated by the Sullivan method [11]. The correlation coefficient between the precise HE65 and estimated HE65 predicted by the formula calculated from prefectural data was 0.976 for men and 0.956 for women.

Municipal LE65 was drawn from the municipal life-table in 2000, using the number of deaths during 1999–2001 and the 2000 census population, and the empirical Bayesian method was applied [24]. The number of those certificated as needing nursing care for LTCI by insurance union in May, 2002, was obtained from the database published by the Welfare and Medical Statistical Agency [25]. SCR was calculated using the municipal population and the age-specific certification rates of the national data from the All-Japan Federation of National Health Insurance Organizations, in which the age categories were 65–69, 70–74, 75–79, and 80+ years [23]. Although most municipalities (N = 2823, 84.0%) had their own insurance unions, there were 61 insurance alliances managed with neighbouring municipalities. In these cases, SCR of the alliance was calculated and used as that of the municipalities for HE estimation.

There was substantial variation in the population size among municipalities, ranging from only a few hundred to one million, and the certification rate in municipalities with a small population size showed extreme fluctuation. To correct the fluctuation of SCR, Bayesian SCR was estimated using hierarchical Poisson regression and Markov chain Monte Carlo (MCMC) method [26-31]. The levels of hierarchy were municipalities for the lower level and secondary medical care zones (SMCZs) for the higher level. The details of calculation of Bayesian SCR are described in the additional file [see Additional file 1]. The range of SCR was decreased from crude SCR of 24.8 to 178.0 (S.D. = 18.3) to Bayesian SCR of 46.8 to 161.3 (S.D. = 16.5).

The regression formula obtained from prefectural data and used for estimation of municipal HE65 was HE65 = 4.772 + 0.706 × LE65 – 1.776 × SCR for men and HE65 = 12.024 + 0.453 × LE65 – 4.576 × SCR for women. Statistical analysis was conducted using SPSS 11.0 for linear regression analysis and MLwiN 1.10 for Bayesian hierarchical Poisson regression. Geographic mapping of HE was conducted using ArcGIS 8.3, with the Japanese map (geographic coordinate system: GRS 1980) obtained from ESRI Japan .

### Analysis of relation to socio-demographic indicators

The socio-demographic indicators used in this study were population, population density, proportion of elderly (aged 65 years or over), percentage of households of a single elderly person (aged 65 years or over), percentage of nuclear households, divorce rate, per capita income, and unemployment rate. The data were drawn from the database based on the population census and other national surveys of 1999 and 2000 [32]. Since crude divorce rate showed a large difference in variance within the three categories of municipalities according to the population size mentioned later, Bayesian method with hierarchical Poisson regression was applied to predict the value adjusted for the statistical fluctuation due to small population size, as well as SCR. A summary of the socio-demographic indicators is shown in Table 1.

**Table 1:** Characteristics of socio-demographic indicators among municipalities in Japan (N = 3361).

Indicator	Mean ± S.D.	(range)
Population (thousands)	37.8 ± 77.3	(0.2 – 1024.1)
Population density (per square kilometer)	928.9 ± 2257.6	(1.5 – 19854.1)
Proportion of elderly (%) ^a^	23.7 ± 7.3	(7.6 – 50.6)
Percentage of households of a single elderly (%)^a^	8.0 ± 4.2	(0.7 – 31.6)
Percentage of nuclear households (%)	54.4 ± 8.7	(20.2 – 78.8)
Divorce rate (per 1000)	1.7 ± 0.4	(0.9 – 4.0)
Per capita income (thousand yen)	1225 ± 299	(419 – 3926)
Unemployment rate (%)	3.88 ± 1.64	(0.0 – 18.1)

^a^65 years or over

The relation of HE65 and LE65 to socio-demographic indicators was examined using correlation coefficient (Spearman's) for total municipalities. Then, we examined the correlation according to the population size of municipalities, in which municipalities were divided into three categories according to population size: less than 10,000 (small, N = 1554), 10,000 to 100,000 (medium-size, N = 1469), and more than 100,000 (large, N = 338). Statistical analysis was conducted using SPSS 11.0.

## Results

### Estimated health expectancy

Table 2 shows LE65 and estimated HE65. HE65 in men (years) ranged from 13.13 to 17.39 with a mean (S.D.) of 15.53 (0.52), and HE65 in women (years) ranged from 14.84 to 20.53 with a mean (S.D.) of 18.01 (0.77). For both men and women, large municipalities showed significantly (p < 0.001) lower HE65 than small and medium-size municipalities. HE65 for the overall Japanese population, which was estimated using the regression formula, was 15.39 for men and 17.62 for women, while HE65 estimated by the Sullivan method with sex- and age-specific long-term care prevalence was 15.40 for men and 17.62 for women, and LE65 in 2000 was 17.56 for men and 22.46 for women [22].

**Table 2:** Life expectancy at 65 years (LE65) and health expectancy at 65 years (HE65) among municipalities in Japan.

Sex	Population size ^a^	LE65		HE65	
			
		Mean ± S.D.	(range)	Mean ± S.D.	(range)
Men	Total	17.56 ± 0.58	(14.9 – 20.3)	15.53 ± 0.52	(13.13 – 17.39)
	Small	17.61 ± 0.59	(15.0 – 19.8)	15.56 ± 0.53	(13.63 – 17.39)
	Medium	17.46 ± 0.58	(15.8 – 20.3)	15.53 ± 0.51	(13.48 – 17.08)
	Large	17.58 ± 0.57	(14.9 – 19.5)	15.42 ± 0.49	(13.13 – 16.84)
Women	Total	22.54 ± 0.68	(20.1 – 27.2)	18.01 ± 0.77	(14.84 – 20.53)
	Small	22.64 ± 0.66	(20.1 – 26.3)	18.03 ± 0.78	(14.83 – 20.53)
	Medium	22.48 ± 0.72	(20.2 – 27.3)	18.09 ± 0.77	(15.45 – 20.11)
	Large	22.40 ± 0.57	(20.6 – 24.5)	17.62 ± 0.62	(14.84 – 18.94)

^a ^Population size: total = total municipalities (N = 3361); small = less than 10,000 (N = 1554), medium = 10,000 to 100,000 (N = 1469), large = more than 100,000 (N = 338)

The geographic mapping of HE65 in men is shown in Figure 1. The central part of the mainland showed accumulation of municipalities with longer HE65, while the northern part of the mainland showed accumulation of municipalities with shorter HE65. Metropolitan areas, especially Osaka, showed relatively shorter HE65. Other regions such as Hokkaido, Kyushu and Shikoku showed a miscellaneous pattern of municipalities with shorter and longer HE65. The geographical pattern in women, shown in Figure 2, was similar to that in men, and the shorter HE65 in the metropolitan areas was more remarkable.

### The relation to socio-demographic indicators

Table 3 shows the correlation coefficients of LE65 and HE65 with socio-demographic indicators. As the number of samples was extremely large, most of the correlation coefficients showed statistical significance (p < 0.001). Compared to LE65, HE65 showed a stronger correlation with the percentage of households of a single elderly person and per capita income for men, and with the percentage of nuclear households, divorce rate and unemployment for men and women, while a weaker correlation with the proportion of elderly for men and women. The correlation with the percentage of households of a single elderly person and per capita income for women was reversed between LE65 and HE65.

**Table 3:** Correlation of health expectancy at 65 years with socio-demographic indicators. Correlation coefficient of life expectancy at 65 years (LE65), health expectancy at 65 years (HE65) with socio-demographic indicators by size of municipalities^a ^in Japan.

	Men					Women				
Indicator	LE65	HE65				LE65	HE65			
	
	Total	Total	Small	Medium	Large	Total	Total	Small	Medium	Large
Population	-0.11	-0.10	-0.12	-0.08	0.05	-0.18	-0.09	0.00	-0.13	-0.06
Population density	-0.11	-0.10	-0.14	-0.03	-0.02	-0.20	-0.10	-0.01	-0.10	-0.27
Proportion of elderly	0.10	0.05	0.08	-0.02	-0.27	0.20	0.05	0.01	0.02	-0.15
Percentage of households of a single elderly	0.00	-0.18	-0.14	-0.27	-0.43	0.17	-0.25	-0.22	-0.33	-0.50
Percentage of nuclear households	-0.01	-0.12	-0.13	-0.09	0.05	-0.03	-0.20	-0.21	-0.23	0.06
Divorce rate	-0.17	-0.26	-0.25	-0.26	-0.44	-0.15	-0.29	-0.29	-0.26	-0.44
Per capita income	0.06	0.17	0.29	0.22	0.46	-0.15	0.16	0.34	0.21	0.26
Unemployment rate	-0.28	-0.35	-0.36	-0.35	-0.52	-0.20	-0.33	-0.24	-0.38	-0.57
Life expectancy at 65 year for some sex		0.80	0.82	0.79	0.86		0.21	0.24	0.17	0.30

^a ^Total = total municipalities (N = 3361); Small = municipalities with a population of less than 10,000 (N = 1554); Medium = municipalities with a population of 10,000 to 100,000 (N = 1469); Large = municipalities with a population of more than 100,000 (N = 338).

Statistical significant: p < 0.001 for coefficients of more than 0.05 or less than 0.05 in total municipalities and for coefficients of more than 0.1 or less than 0.1 in small, medium, and large municipality groups.

Concerning the correlation coefficients according to population size of municipalities, the socio-demographic indicators showed a stronger correlation in large municipalities (with a population of more than 100,000) than in small and medium-size municipalities, except for population, population density, and the percentage of nuclear households. Especially, the percentage of households of a single elderly person, divorce rate, per capita income and unemployment rate showed correlation coefficients of more than 0.4 (or less than -0.4) in large municipalities.

## Discussion

This study estimated HE of older people and demonstrated its relation to socio-demographic indicators at the municipal level across Japan. As a result, the estimated HE65 (years) ranged from 13.13 to 17.39 for men and from 14.84 to 20.53 for women, and it was closely related to area socio-demographic conditions such as the percentage of households of a single elderly person, divorce rate, per capita income and unemployment rate. The relation of HE65 to socio-demographic indicators was stronger than that of LE65, and municipalities with a larger population showed a stronger relation between HE65 and socio-demographic factors.

Lower income and education are strongly related to higher morbidity of older people and shorter LE and HE in other countries [13,15,33]. In a previous study in Japan, however, area indicators related to income, education and unemployment showed little association with mortality of older people [26]. This finding was consistent with the result of this study that LE65 showed a significant but weak correlation with socio-demographic indicators. HE65 was more strongly correlated with socio-demographic indicators than LE65. These findings indicated that the health status measured by not only mortality but also morbidity is more sensitive for area socioeconomic conditions than that measured by only mortality, and the health level of older people would be substantially decreased by socioeconomic conditions in disadvantaged areas.

A notable finding of this study was the negative relation between HE65 and the percentage of households of a single elderly person and divorce rate. Shorter life expectancy might cause an increase in older people living alone because of an increase in widows/widowers. However, considering that the proportion of a single elderly person did not showed a negative relation to LE65, it is likely that the shorter HE was caused by an increase of older people living alone. It was confirmed by previous studies that living alone including divorce and loss of a spouse has a negative impact on the health of older people [16]. In addition, since most divorced persons were in the young- and middle-aged population, and divorces among older people are more unusual than among the middle-aged population [34], it seems plausible that the increased divorce rate influence the health status of older people through decreased support in families and communities.

In this study, we found a difference in the relation between HE65 and socio-demographic factors according to the size of municipalities. A previous study, also, showed that urban areas showed a stronger relation between health status and area socioeconomic conditions than rural areas [35]. The first possible reason for this urban-rural difference is the choice of indicator/index for area socioeconomic conditions. A previous study demonstrated that the traditional deprivation index (Townsend index) was related to health only in urban areas, while an index including more multiple domains was related to health in both urban and rural areas [19]. The second possible explanation is the existence of other factors mediating the relations, such as the social support network. The social support network has been demonstrated to influence the health status, and less social support is closely linked to poorer health in older people [12,36]. Also, social factors are less associated with mortality in a socially cohesive area [37], and the relation between socioeconomic inequality and mortality is mediated by so-called social capital [38]. Since urban areas show less social support network than rural areas in Japan [39,40], it is likely that in urban areas, less social support network causes a decline in health status and enhances the influence of other social factors on health status in the elderly population.

A few weaknesses of this study should be mentioned, especially concerning the estimation of disability prevalence and HE. First, the disability prevalence used for HE estimation was based on LTCI data. As a previous study demonstrated, utilization of LTCI services was dependent on not only the level of disability but also socioeconomic conditions such as household income and family structure [41]. However, LTCI in Japan provides consistent insurance across the nation and universally covers all elderly persons aged over 65 years [42], and thus, the influence of socioeconomic conditions on service utilization and regional variation would seem to be relatively small. In addition, LTCI data are the sole source for routine estimation of HE at present. Second, the disability prevalence (SCR) was estimated by hierarchal regression and MCMC procedure (fully Bayes method) [27-31]. Bayes estimation is commonly used for data smoothing in small area analysis and disease mapping, while alternative methods including empirical Bayes estimation can be applied [29-31,43-46]. Although SMCZ is generally used as a higher-level regional unit for Bayes estimation of municipal mortality data in Japan [24,26,47,48], an alternative hierarchical setting of regional levels would result in different figures of SCR, and consequently of HE. Finally, HE65 was estimated by a simple linear regression formula based on prefectural data. The validity of the estimated HE was confirmed by the municipal data of one prefecture, and the correlation coefficients between the precise and estimated HE were large enough to use the estimated HE for geographical comparison, instead of the precise HE. However, the application of this HE estimation for individual municipalities should be thought out. To expand the use of HE, the routine publication of detailed data of LTCI including the sex- and age-specific number of those certificated as needing nursing care is expected, which would enable calculation of the precise HE.

The results of this study have several implications in prolonging healthy longevity and supporting older people. The strong association between HE65 and socio-demographic factors indicates the need for efforts in the social context. Especially, there should be a focus on older people living alone. Also, the strong relation between HE65 and socio-demographic factors in large municipalities indicates that older people living in urban areas lack a social support network and are vulnerable to socioeconomically disadvantaged conditions. The social support network including an informal network and community activities will contribute to improving the health status of older people and prolongation of healthy longevity, especially in urban areas.

Although we addressed HE in this study, the results suggest that disability prevalence (SCR) is a useful indicator independently of mortality data including LE. Since HE was estimated using LE and SCR, the difference in the relation with socio-demographic indicators between LE and HE implies that SCR includes quite different dimensions from LE. The use of long-term care prevalence, in combination with mortality indicators (e.g., LE) and comprehensive indicators (e.g., HE), will contribute to concrete health policy making and evaluation for improvement of health, well-being, and quality of life in elderly population.

## Conclusion

This study established a method to estimate HE available at the local level using a linear regression formula with life expectancy and long-term care prevalence. Municipal HE65 (years) ranged from 13.13 to 17.39 for men and from 14.84 to 20.53 for women. As a result of correlation analysis, a decreased HE was associated with higher percentage of households of a single elderly person and divorce rate, and lower socioeconomic conditions. This study suggests that older people in urban areas are susceptible to socio-demographic factors, and a social support network for older people living in socioeconomically disadvantaged conditions should be encouraged.

## Competing interests

The author(s) declare that they have no competing interests.

## Authors' contributions

YF designed the study, analyzed the data, and drafted the article. KN helped to interpret the results and edited the draft. TT supervised the data analysis and writing the article.

## Pre-publication history

The pre-publication history for this paper can be accessed here:



## Supplementary Material

Additional File 1 **Calculation of Bayesian SCR**. Calculation of standardized certification ratio (SCR) using Bayesian hierarchical Poisson regression Click here for file
