# Supplementary material for: Municipal health expectancy in Japan: decreased healthy longevity of older people in socioeconomically disadvantaged areas
Source: BMC Public Health. 2005 Jun 14;5:65. doi: 10.1186/1471-2458-5-65 (PMC1177965; doi:10.1186/1471-2458-5-65)
Supplement: Additional File 1 — Calculation of Bayesian SCR. Calculation of standardized certification ratio (SCR) using Bayesian hierarchical Poisson regression [file 1471-2458-5-65-S1.pdf]

Additional file 1

Calculation of standardized certification ratio (SCR) using Bayesian hierarchical Poisson regression

Objective

There is substantial variation in the population size among municipalities (cities, towns, and villages) in Japan, ranging from only a few hundred to one million, and the certification rate for long-term care insurance (LTCI) in municipalities with a small population size is expected to show extreme fluctuation. This calculation aimed to correct the fluctuation of standardized certification ratio (SCR) for LTCI, using Bayesian hierarchical Poisson regression [S1-S4].

Methods

The basic units of the SCR calculation were municipalities, since each municipality has an insurance union (N=2816). Besides, there are insurance alliances (N=61) managed with neighboring municipalities. In these cases, the units included multiple municipalities.

The secondary medical care zone (SMCZ) was used as a higher level in the Bayesian hierarchical regression model. Each prefecture was divided into multiple SMCZs defined by prefectural governments for medical care planning according to the Medical Service Law. There were 360 SMCZs across Japan in 2000, each of which consisted of neighboring municipalities and covered a population of 300,000 on average. SMCZ was defined in consideration of the area of the resident's daily life, and medical care planning including resource allocation of primary to tertiary health care was conducted based on this zone. It was confirmed by previous studies that SMCZ is suitable for the prior distribution in estimation of empirical Bayes standardized mortality ratio of municipalities [S5,S6].

For municipality *i* of SMCZ *j*, we modelled the observed number of certificated persons O_{ij} as a Poisson realization:

$$O_{ij} \sim \text{Poisson}(\pi_{ij})$$

$$\log(\pi_{ij}) = \log(E_{ij}) + \beta + v_j + u_{ij}$$

$$v_j \sim \text{N}(0,\sigma_v^2),\ u_{ij} \sim \text{N}(0,\sigma_u^2)$$

where π_{ij} and E_{ij} are the predicted and expected number of certificated persons, respectively, and the intercept includes the grand intercept, β, varying across SMCZ and municipalities via random effects v_j and u_{ij}, respectively [S1,S2,S9]. The observed number of certificated persons by municipality was obtained from the database of the Welfare and Medical Service Agency [S7]. The expected number of certificated persons was calculated from the population aged over 65 of the unit and the age-specific certification rates of the national data, in which the age categories were 65-69, 70-74, 75-79, and 80+ years [S8]. Parameters were estimated using Markov chain Monte Carlo (MCMC) procedure [S9]. The starting values of the simulation were given by the iterative generalized least square (IGLS), and for the Bayesian calibration of the model non-informative priors for the fixed parameter and variance terms were provided as follows: $p(\beta) \propto 1$ and $p(1/\sigma_v^2)$, $p(1/\sigma_u^2) \sim \text{Gamma}(\varepsilon,\varepsilon)$, where ε was very small [S10]. The simulation was performed by Gibbs sampling with a burn-in of 500 iterations followed by 5000 further iterations. Then, Bayesian SCR was predicted using the regression equation [S1,S2]. The analysis was conducted using MLwiN 1.10 [S10].

Results

The crude and Bayesian SCR according to population size are shown in Table S1, with the data by the size of municipalities. The crude SCR for total municipalities ranged from 24.8 to 178.0 with standard deviation

(SD) of 18.3, while the Bayesian SCR ranged from 46.8 to 161.3 with SD of 16.2. The decrease in the variation of Bayesian SCR compared to the crude SCR was pronounced for municipalities with smaller population: SD among these municipalities with a population less than 10,000 (N=1303) changed from 20.1 for the crude SCR to 16.3 for the Bayesian SCR.

Table S1 Crude and Bayesian standardized certification ratio (SCR) for long-term care insurance by municipality in Japan.

	Crude SCR		Bayesian SCR	
Size of municipalities [a]	Mean±SD	(range)	Mean±SD	(range)
Total	90.5±18.3	(24.8-178.0)	90.5±16.2	(46.8-161.3)
Small (N=1303)	91.5±20.1	(24.8-178.0)	91.5±16.3	(52.4-161.3)
Medium (N=1621)	88.2±17.0	(40.3-149.1)	88.2±16.3	(46.8-147.6)
Large (N=221)	97.0±13.3	(64.5-151.7)	96.9±13.2	(64.7-151.3)

[a] Small = municipalities with a population of less than 10,000; Medium = municipalities with a population of 10,00 to 100,000; Large = municipalities with a population of more than 100,000.

References

S1. Leyland AH, McLeod A: **Mortality in England and Wales, 1979-1992.** Glasgow: MRC Social & Public Health Sciences Unit; 2000. http://www.msoc-mrc.gla.ac.uk/Publications/pub/occasional_MAIN.html

S2. Fukuda Y, Nakamura K, Takano T: **Municipal socioeconomic status and mortality in Japan: sex and age differences, and trends in 1973-1998.** *Soc Sci Med* 2004, **59**:2435-2445.

S3. Nakaya T: **Spatial epidemiology**. In *Health GIS*. Edited by Nakaya T, Tanimura S, Nihei N, Horikoshi Y. Tokyo: Kokon Shoin; 2004:74-121.

S4. Best N, Richardson S, Thomson A: **A comparison of Bayesian spatial models for disease mapping**. *Stat Methods Med Res* 2005, 14:35-59.

S5. Nakaya T: **An information statistical evaluation of areal units for mortality mapping: toward constructing an analyzing system of disease maps on geographical information system**. *Theory and Applications of GIS* 1996, **4**:53-60.

S6. Hirako T, Sakeki N, Nakaya T: **Application of Bayesian methods on municipal health statistics**. *Koseino Shihyo* 1999, **46(10)**:3-11.

S7. Ministry of Health, Labour and Welfare: **Prefectural life table 2000.** Tokyo: Health and Welfare Statistics Association; 2003.

S8. All-Japan Federation of National Health Insurance Organizations: **Statistics for the long-term care insurance.** Tokyo: All-Japan Federation of National Health Insurance Organizations; 2002. http://www.kokuho.or.jp/shiryou/index.htm.

S9. Wakefield JC, Best NG, Waller L: **Bayesian approaches to disease mapping**. In *Spatial Epidemiology*. Edited by Elliot JC, Wakefield NG, Best NG, Briggs DJ. New York: Oxford University Press; 2000:104-127.

S10. Rashash J, Browne W, Goldstein H, Yang M, Plewis I, Healy M, Woodhouse G, Draper D, Langford I, Lewis: **A user's guide to MLwiN**. London: Centre for Multilevel Modelling, Institute of Education, University of London; 2002.
